# Data Resource Profile: COVerAGE-DB: a global demographic database of COVID-19 cases and deaths

**DOI:** 10.1093/ije/dyab027

**Published:** 2021-05-15

**Authors:** Tim Riffe, Enrique Acosta, the COVerAGE-DB team, Enrique José Acosta, Diego Manuel Aburto, Anna Alburez-Gutierrez, Ainhoa Altová, Ugofilippo Alustiza, Simona Basellini, Didier Bignami, Eungang Breton, Jorge Choi, Gonzalo Cimentada, Emanuele De Armas, Alicia Del Fava, Viorela Delgado, Jessica Diaconu, Christian Donzowa, Antonia Dudel, Alain Fröhlich, Mariana Gagnon, Victor Garcia-Crisóstomo, Armando M Garcia-Guerrero, Irwin González-Díaz, Dagnon Hecker, Marina Eric Koba, Mine Kolobova, Mélanie Kühn, Chia Lépori, Andrea Liu, Mădălina Lozer, Lilian Manea, Muntasir Marey, Ryohei Masum, Céline Mogi, Saskia Monicolle, Ronald Morwinsky, Mikko Musizvingoza, Marília Myrskylä, Michelle R Nepomuceno, Natalie Nickel, Anna Nitsche, Samuel Oksuzyan, Emmanuel Oladele, Oluwafunke Olamijuwon, Soumaila Omodara, Mariana Ouedraogo, Marius Paredes, Manuel D Pascariu, Raquel Piriz, Larbi Pollero, Federico Qanni, Filipe Rehermann, Silvia Ribeiro, Francisco Rizzi, Adil Rowe, Isaac R Sarhan, Erez Sasson, Jiaxin Shomron, Rafael Shi, Cosmo Silva-Ramirez, Catalina Strozza, Sergi Torres, Fumiya Trias-Llimos, Alyson Uchikoshi, Paola van Raalte, Estevão Vazquez-Castillo, Muhammad A Vilela, Iván Ali Waqar, Virginia Williams, Zarulli

**Affiliations:** Laboratory of Population Health, Max Planck Institute for Demographic Research, Rostock, Germany

## Data resource basics

Information about pandemic dynamics is crucial to understand the potential impacts on populations, design mitigation strategies and evaluate the efficacy of their implementation. Centralization, standardization and harmonization of data are critical to enable comparisons of the demographic impact of COVID-19 which take into account differences in the age and sex compositions of confirmed infections and deaths. The international data landscape must keep pace with the global march of the pandemic, and researchers must work to triangulate the available data to create comparable measures to monitor and predict its demographic impacts.

COVerAGE-DB aims to provide global coverage of key demographic aspects of the COVID-19 pandemic as it unfolds in an up-to-date, transparent and open-access format. COVerAGE-DB offers data with standardized count measures by sex and harmonized age groups, which is a necessary but not sufficient condition to allow comparisons between populations at national and subnational scales.

**Figure 1:**
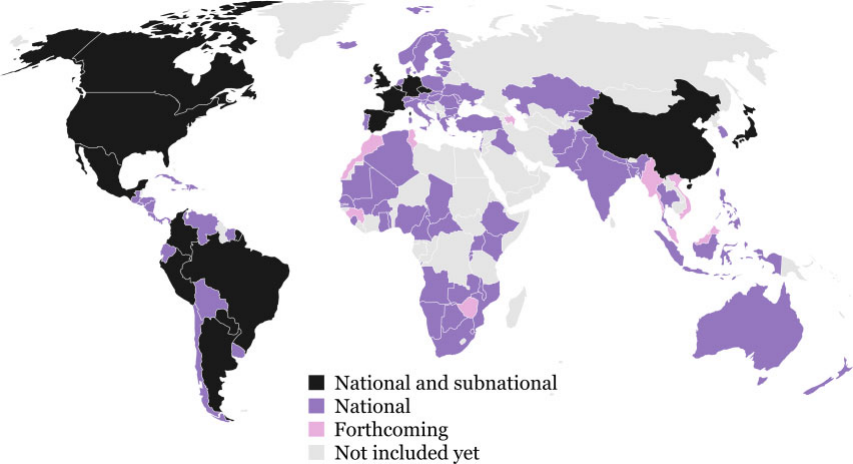
Availability of national and subnational information on COVID-19 cases, deaths, and tests in the countries included in the database as of 7 January, 2021.

The database is currently under expansion through both the increase in coverage of national and subnational populations and the inclusion of more recent periods as the pandemic continues. At the time of writing, the database contains daily counts of COVID-19 cases, deaths and tests performed, by age and sex, for 108 national and 371 subnational populations around the world, depending on the available data for each source. The date range available for each country or subpopulation varies. In several country series, the database includes the earliest confirmed cases in January 2020. For most populations, the database includes daily time series, beginning from an initial starting date when the data were first released or collected by our team. Figure 1 displays a map of countries included in the database, indicating at least one subnational population from 13 countries. A detailed overview of data availability is given in a searchable table: [https://bit.ly/3kVDrLD].

## Data collected

Official counts of COVID-19 cases, deaths, and tests are extracted from reports published by official governmental institutions, such as health ministries and statistical offices. Depending on the source, data are collected in a variety of formats, including machine-readable files, pdf tables, html tables, interactive dashboards, press releases, official announcements via Twitter, and in a few instances, from digitized graphics. A full list of data sources is available in a dashboard view [https://bit.ly/2Qg1MxL].

Generally, COVID-19 cases, deaths and tests in age groups are reported as counts, but some sources report data in other metrics (fractions, percentages, ratios) or as summary indicators such as case fatality ratios (CFRs) by age. Reported age intervals vary by source, ranging from single ages to 30-year or greater age bands, and sometimes reported age intervals change over time within sources. Usually data are reported as cross-sectional snapshots of cumulative counts, but some sources give full time series of new cases or deaths, in which case we cumulate counts over time. We also collect standard metadata on each of the sources to capture various characteristics of the collected data, such as the primary collection channels, definitions used and notes on major disruptions or events. An overview of key fields from these metadata is shared as a spreadsheet [https://bit.ly/2FAmKFn].

## Data production

All source data are entered into standard spreadsheet templates hosted in a central folder on Google Drive. Data entry into the templates is either manual or automatic, depending on the source.

R programs collect data from the source templates and compile the merged input database. The merged input file is then subject to a series of automatic validity checks. Initial checks are carried out by the individual responsible for data collection and entry, using an interactive application [https://mpidr.shinyapps.io/cleaning_tracker/]. Data are then harmonized to standard metrics (counts), measures (cases, deaths, tests) and age bands (5- and 10-year age intervals). Harmonization procedures include rescaling to ensure coherence between age distributions and reported total counts. Age group harmonization is done using the penalized composite link model for ungrouping^1^ which was designed for splitting histograms of count data. Output data also include a file containing selected diagnostics of data quality, such as completeness of age reporting, for each source and date.

The complete details on all steps of production are available in the COVerAGE-DB Method Protocol, which is publicly available on the web.^2^ A table listing which adjustments are applied to each population is available on the project website [https://bit.ly/2E61BSV]. The merged input database, the harmonized output and the data quality files are uploaded daily as zipped csv files to an Open Science Framework repository (OSF) [https://osf.io/mpwjq/]. A GitHub repository [https://bit.ly/2YbtPCJ], which is linked to OSF, contains all R scripts used in the complete production pipeline, including compilation, diagnostics and harmonization.

## Data resource use

Since collection efforts began for COVerAGE-DB in late March 2020, we are aware of 15 studies using the data, many of which provide R code online and are fully reproducible. Broadly, these studies aim to measure the influence of demographic factors on mortality from COVID-19,^3^^,^^4^ assess the pandemic impact on health and mortality within^5^^,^^6^ and across populations,^7–12^ analyse COVID-19 data availability and quality,^13^ propose methodological innovations that allow comparisons of CFRs^14^ and the development of indirect methods to estimate infections in the population.^15^^,^^16^ The database is also used to monitor COVID-19 impacts in particular age ranges. For instance, UNICEF has used the database for monitoring the burden of the pandemic on children around the world^17^ and the UN Department of Economic and Social Affairs has used it similarly to focus on older age groups.^18^

**Figure 2:**
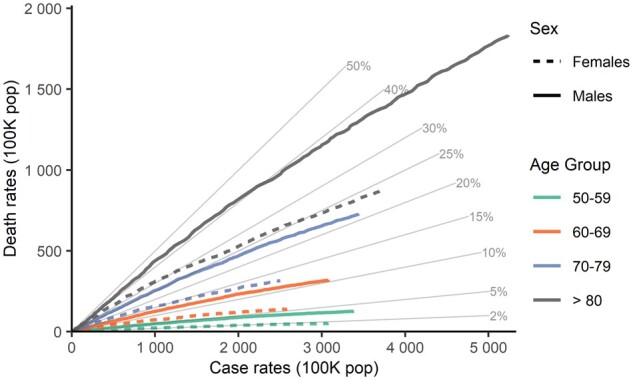
Relationship between deaths and cases per 100 000 population by age group and sex in Colombia, until 7 November 2020. Diagonal lines indicate the case fatality ratio.

As an example of the analyses that COVerAGE-DB enables, Figure 2 displays changes in the relation between age-specific deaths and cases rates in Colombia, inspired by Figure 1 of Dudel *et al*.^14^ We divide both cases and deaths in each age band by the respective population sizes. Diagonal lines indicate age-specific CFRs. The graph illustrates a sharp increase in CFR over age for each sex, and displays considerable sex differences. For instance, men aged 60–69 in Colombia have almost the same CFR (approximately 12% risk of death after COVID-19 disease diagnosis) as women aged 70–79.

**Figure 3:**
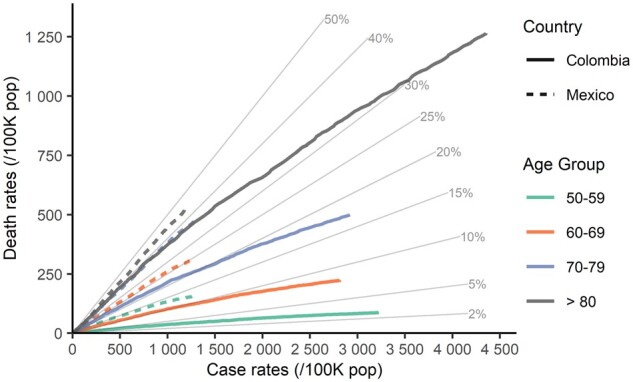
Relationship between deaths and cases per 100 000 population by age group in Mexico and Colombia, until 7 November 2020. Diagonal lines indicate the case fatality ratio.

We repeat this exercise to compare Colombia with Mexico (see Figure 3), where standardizing by population size is more justified. CFRs and death rates are much higher in Mexico than in Colombia in each age band—around 2-fold—except for ages 80+, which show a substantial reduction in the CFR difference, and much higher death rates for Colombia.

This comparison between Colombia and Mexico allows us to illustrate several issues in data quality to be considered when comparing COVID-19 outcomes between populations in general. Besides the economic and sanitary conditions that make Latin American countries more vulnerable to the pandemic, the lack of unambiguous definitions of COVID-19 cases and deaths and the limited testing capacity represent major challenges for data quality assessment.^19–21^ We focus here on definitions and testing strategies.

With respect to COVID-19 case and death definitions, criteria have varied since records started. At the time of data retrieval, both countries use laboratory, clinical and epidemiological criteria to confirm SARS-CoV-2 infections.^22^^,^^23^ However, the vast majority of COVID-19 cases and deaths are confirmed with RT-PCR tests results in both populations (99.6% and 91.6% in Colombia and Mexico, respectively).^24^^,^^25^ Regarding the definition of tests, whereas in Colombia it refers to laboratory samples tested (4.5 M as of 7 November 2020), in Mexico it alludes to persons (2.3 M). Because individuals may be tested more than once, comparison between these two units is not straightforward. Testing performance measures, such as positive rates (e.g. 30% in Colombia and 45% in Mexico^26^), are essential for interpreting differences in cases and deaths across populations, because they help to assess the extent of infection under-reporting.^27^ However, differences in test definitions pose serious challenges for direct comparisons. Dates in both sources are comparable, corresponding to the occurrence of events. Since information from both sources relies on individual-level databases, delays in diagnosis and death registration are retrospectively adjusted.

Differences in testing capacity and strategy between countries are also key determinants for infection diagnosis. Given both the magnitude of contagion and limited resources in the region, Latin American countries have struggled to increase testing capacity proportionally to the spread of the infection.^28^^,^^29^ Although with very limited capacity, the testing approach of Colombia has been to test as many suspected cases as possible. In contrast, an important part of the test strategy in Mexico has focused on inferring the extent of contagion in the population by using nationally representative samples (known as Centinela, which represent 36.5% of all confirmed infections at the date under observation), and it has gradually included a small proportion of suspected infections outside the Centinela system.^23^ On 7 November 2020, Colombia performed five times more tests per capita than Mexico. These differences in testing regimes between both countries may account for a substantial part of the CFR discrepancies observed in Figure 3.

The differences in definitions and testing strategies between populations highlight challenges in making comparisons and also the need to produce data with sufficient detail to adjust for biases. For this reason, alongside data on cases, deaths and tests, COVerAGE-DB offers additional information on metadata and quality metrics that are needed for a cautious interpretation of the data and their limitations. It is our view that researchers should triangulate creatively from all available data rather than avoid difficult comparisons.

## Strengths and weaknesses

Since the beginning of the pandemic, it has been evident that population characteristics are key to understanding the prevalence, spread and fatality of COVID-19 across countries. However, data on cases, deaths and tests disaggregated by age and sex are not easily comparable across countries, and sometimes not even accessible. The main strength of COVerAGE-DB is to provide a centralized, open-access and fully reproducible repository of age-and sex-specific case, death and test counts from COVID-19, collected from official sources and harmonized to standard output formats. The data harmonization process is transparent, following a strict protocol.^2^ The initial input data are provided alongside the harmonized counts, as well as the code used to harmonize the different input measures, metrics and age groups into comparable granular output metrics. All scripts are written in the open-source R programming language.^30^ The data sources and limitations are documented for each country in a standard metadata framework.

A limitation of the COVerAGE-DB is the heterogeneous and difficult-to-evaluate quality of the underlying data. No single data source can currently claim accurate estimates of COVID-19 incidence or fatalities. Age-specific case counts are highly dependent upon the testing capacity,^31^ testing strategy^32^ and differences in the definition of cases across sources and over time. Recorded cases underestimate infections everywhere, with underestimation expected to vary by age, given the relationship between age and case severity.^33^ The accuracy of diagnostic RT-PCR tests used to confirm infections is also known to vary.^34^ Furthermore, at any given date, cumulative counts are underestimated because of the lag between infection and a positive test result.^35^

Death counts from COVID-19 are also likely underestimated for similar reasons and also due to various kinds of delays in death registration. Media reports have circulated about intentional data manipulation in some of the official data covered in the database.^36^ Excess all-cause mortality has been observed across many regions.^37–40^ Although some of these deaths likely are from postponing or foregoing treatment from non-COVID-19-related causes, the magnitude of this excess is suggestive that numerous COVID-19-related deaths are classified under different causes. Populations also differ in whether deaths of suspected COVID-19 cases are included in official statistics and in post-mortem practices when an infection is suspected.^41^ Some populations only report deaths occurring in hospitals, neglecting a potentially sizeable proportion of deaths occurring in institutional settings and at home.^42^ Most populations currently report all deaths to confirmed SARS-CoV-2 infections as COVID-19 deaths for this database, but the underlying cause of death eventually reported on the death certificate may differ in patients with severe comorbidities. To mitigate biases and misinterpretations due to different practices and definitions, such information is constantly updated and documented in the metadata of the database which are freely accessible to users. Further, a supplementary data quality metrics file contains a suite of data quality indicators that is easily merged with the main output data. Quality metrics include age-reporting completeness, some indicators on how aggressive age harmonization is, and two positivity measures from Our World in Data database on COVID-19 testing.^26^

All of these issues compromise the comparability of the data contained within the COVerAGE-DB, both across populations at any given time and within populations over time. That is, the database enables direct calculation of age-specific CFRs, but one must be careful when making comparisons. Care must also be taken not to interpret calculated CFRs as infection fatality ratios, the latter of which include both detected and undetected SARS-CoV-2 infections in the denominator. Proper estimation of incidence and fatality, and of total demographic impacts, will likely require triangulating data across numerous sources as these become available. To this end, the COVerAGE-DB was designed to be easily merged with other databases such as the Our World In Data testing or excess mortality data,^26^ the COVID-19 dashboard of Johns Hopkins,^43^ the World Population Prospects database^44^ and the Short Term Mortality Fluctuations database.^40^ Moreover, given that we have near-complete time series capturing the whole pandemic curve in some places, careful modelling of lag structures might allow some of these data-driven biases to be estimated.

## Data resource access

**Figure 4:**
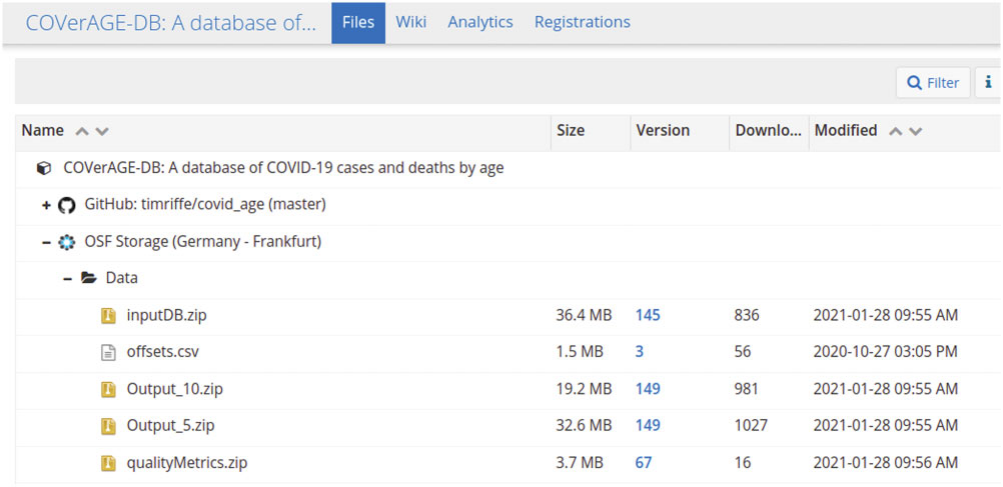
View of the Open Science Framework (OSF) repository, File section [https://osf.io/mpwjq/files/]. To download data files, click on Data, and select one of the files.

Both merged input and harmonized output files can be downloaded directly from the OSF site [https://osf.io/mpwjq doi: 10.17605/OSF.IO/MPWJQ, which contains a folder called ‘Data’ with four files of primary data. Figure 4 shows where to find the files in the OSF repository.

Each of the main data files has a stable link (see Table 1) which always points to the most recent version. Each file is a zipped csv file by the same name. For stable links to download particular versions, click on the version number in the Version column seen in Figure 4. Users can note versions either by referring to timestamps provided in the headers of data files or by referring to OSF file version numbers, which increment with each daily update.

**Table 1:** The main data files, a description of their content, and their stable URLs

Filename	Description	Stable URL
1. inputDB.zip	Data in original metrics, measures and age intervals	[https://osf.io/ 9dsfk/]
2. Output_5.zip	Data with standardized metrics and measures, and harmonized age groups in 5-year intervals	[https://osf.io/ 7tnfh/]
3. Output_10.zip	Data with standardized metrics and measures, and harmonized age groups in 10-year intervals	[https://osf.io/ 43ucn/]
4. qualityMetrics.zip	Selected data quality indicators by location and date	[https://osf.io/ qpfw5/]

A data dictionary is given in both the OSF wiki [https://osf.io/mpwjq/wiki/home/] and the Method Protocol.^2^ Files are shared in csv format to be as universally accessible as possible. A guide to getting started using the data in R is also provided [https://bit.ly/3g8nIVU], to merge COVerAGE-DB with other databases, and tips for other statistical packages may also be added. Users are encouraged to reach out for further information or advice on using the database, or to express interest in the project at: [coverage-db@demogr.mpg.de].

**Profile in a nutshell**

- COVerAGE-DB is an open-access database including cumulative counts of confirmed COVID-19 cases, deaths and tests by age and sex. Original data and sources are provided alongside data and measures in age-harmonized formats.
- The database is in continuous development. It includes data since January 2020, and as of 7 January 2021, it includes 108 countries and 371 subnational areas.
- The database also documents variations in definitions of all input data and indicators of reporting completeness across sources and over time.
- An international team, composed of more than 60 researchers, contributed to the collection of data and metadata in COVerAGE-DB from governmental institutions, as well as to the design and implementation of the data processing and validation pipeline. We encourage researchers interested in supporting this project to send a message to the email: [coverage-db@demogr.mpg.de].

## References

[1] Rizzi S , Gampe J, Eilers PHC. Efficient estimation of smooth distributions from coarsely grouped data. Am J Epidemiol 2015;182:138–47. 26081676 10.1093/aje/kwv020 PMC4493979

[2] Riffe T , Rizzi S, Dudel C et al *Method Protocol for the COVerAGE-DB. Report No. 1.* 2020. https://osf.io/jcnw3/ (28 January 2021, date last accessed).

[3] Medford A , Trias-Llimós S. Population age structure only partially explains the large number of COVID-19 deaths at the oldest ages. Demogr Res 2020;43:533–44.

[4] Arpino B , Bordone V, Pasqualini M. No clear association emerges between intergenerational relationships and COVID-19 fatality rates from macro-level analyses. Proc Natl Acad Sci U S A 2020;117:19116–21. 32699150 10.1073/pnas.2008581117 PMC7431085

[5] Uchikoshi F. COVerAGE-JP: COVID-19 deaths by age and sex in Japan. *SocArXiv*, 19 August 2020. doi:10.31235/osf.io/cpqrt. Preprint: not peer reviewed.

[6] Verdery AM , Smith-Greenaway E, Margolis R, Daw J. Tracking the reach of COVID-19 kin loss with a bereavement multiplier applied to the United States. Proc Natl Acad Sci U S A 2020;117:17695–701. 32651279 10.1073/pnas.2007476117 PMC7395491

[7] Hulíková Tesárková K. Demographic aspects of the COVID-19 pandemic in Italy, Spain, Germany, and South Korea. Geografie 2020;125:139–70.

[8] Kashnitsky I , Aburto JM. COVID-19 in unequally ageing European regions. World Dev 2020;136:105170. 32895594 10.1016/j.worlddev.2020.105170 PMC7455200

[9] Sasson I. Age and COVID-19 mortality: A comparison of Gompertz doubling time across countries and causes of death. *Demogr Res* 2021; **44**:379–96.

[10] Gonzalez-Garcia N , Miranda-Lora AL, Mendez-Galvan J et al International heterogeneity in coronavirus disease 2019 pediatric mortality rates. *Boletín Médico del Hospital Infantil de México* 2021; 78:24–28 10.24875/BMHIM.20000291 33690595

[11] Pifarré I Arolas H , Acosta E, Casasnovas GL et al Years of life lost to COVID-19 in 81 countries. *Sc Rep* 2021; 11. doi: 10.1038/s41598-021-83040-3 10.1038/s41598-021-83040-3 PMC7892867 33603008

[12] Kippen R. Australian age-sex-specific COVID-19 mortality in international comparative perspective, to June 2020. Aust Popul Stud 2020;4:33–38.

[13] Lloyd-Sherlock P , Sempe L, McKee M, Guntupalli A. Problems of data availability and quality for COVID-19 and older people in low- and middle-income countries. Gerontologist 2020; 61:141–144 10.1093/geront/gnaa153 PMC7665497 33017839

[14] Dudel C , Riffe T, Acosta E, van RA, Strozza C, Myrskylä M. Monitoring trends and differences in COVID-19 case-fatality rates using decomposition methods: Contributions of age structure and age-specific fatality. PloS One 2020;15:e0238904. 32913365 10.1371/journal.pone.0238904 PMC7482960

[15] Bohk-Ewald C , Dudel C, Myrskylä M. A demographic scaling model for estimating the total number of COVID-19 infections. Int J Epidemiol 2021;49:1963–71. 33349859 10.1093/ije/dyaa198 PMC7799106

[16] Louca S. COVID-19 prevalence in 161 countries and over time. *medRxiv*, 2 December 2020. doi:10.1101/2020.12.01.20241539. Preprint: not peer reviewed.

[17] UNICEF. *Averting a Lost COVID Generation. A Six-Point Plan to Respond, Recover and Reimagine a Post-Pandemic World for Every Child*. 2020. https://www.unicef.org/reports/averting-lost-generation-covid19-world-childrens-day-2020-brief (28 January 2021, date last accessed).

[18] United Nations Department of Economic and Social Affairs, Population Division. *World Population Ageing 2020 Highlights.* Report No.: ST/ESA/SER.A/451. 2020. https://www.un.org/development/desa/pd/sites/www.un.org.development.desa.pd/files/undesa_pd-2020_world_population_ageing_highlights.pdf (28 January, 2021, date last accessed)

[19] França EB , Ishitani LH, Teixeira RA et al Óbitos por COVID-19 no Brasil: quantos e quais estamos identificando? [Deaths due to COVID-19 in Brazil: how many are there and which are being identified?] Rev Bras Epidemiol 2020;23. doi: 10.1590/1980-549720200053. 10.1590/1980-549720200053 32578810

[20] Rao C. Bulletin of the World Health Organization*. Medical Certification of Cause of Death for COVID-19*. 2020. http://www.who.int/bulletin/volumes/98/5/20-257600/en/ (10 November 2020, date last accessed). 10.2471/BLT.20.257600 PMC7265943 32514191

[21] Peña R. *Y al Tercer Día, Resucitó [And on the third day, he resuscitated]*. 2020 https://www.etcetera.com.mx/opinion/y-al-tercer-dia-resucito/ (10 November 2020, date last accessed).

[22] Instituto Nacional de Salud. *Orientaciones para la Vigilancia en Salud Pública de la Covid19 [Guidelines for Public Health Surveillance of Covid19]*. 2020. http://www.ins.gov.co/Noticias/Coronavirus/Estrategia%20VSP%20COVID-19%2023072020.pdf (10 November 2020, date last accessed).

[23] Secretaría de Salud de México. Dirección General de Epidemiología. *Lineamiento Estandarizado para la Vigilancia Epidemiológica y por Laboratorio de la Enfermedad Respiratoria Viral [Standardized Guidelines for Epidemiological and Laboratory Surveillance of Viral Respiratory Disease].* 2020. https://coronavirus.gob.mx/wp-content/uploads/2020/09/Lineamiento_VE_y_Lab_Enf_Viral_Ago-2020.pdf (10 November 2020, date last accessed).

[24] Ministerio de Salud y Protección Social. *Casos positivos de COVID-19 en Colombia [Positive cases of COVID-19 in Colombia].* 2020. https://www.datos.gov.co/Salud-y-Protecci-n-Social/Casos-positivos-de-COVID-19-en-Colombia/gt2j-8ykr/data (10 November 2020, date last accessed).

[25] Secretaría de Salud. *Base de Datos de COVID-19 - Datos Abiertos Dirección General de Epidemiología [COVID-19 Database - Open Data General Directorate of Epidemiology]*. 2020. http://www.gob.mx/salud/documentos/datos-abiertos-152127 (10 November 2020, date last accessed).

[26] Hasell J , Mathieu E, Beltekian D et al A cross-country database of COVID-19 testing. Sci Data 2020;7:345. 33033256 10.1038/s41597-020-00688-8 PMC7545176

[27] Kigozi SP , Kigozi RN, Sserwanga A et al Malaria burden through routine reporting: relationship between incidence and test positivity rates. Am J Trop Med Hygiene 2019;101:137–47. 10.4269/ajtmh.18-0901 PMC6609187 31074412

[28] Burki T. COVID-19 in Latin America. Lancet Infect Dis 2020;20:547–48. 32311323 10.1016/S1473-3099(20)30303-0 PMC7164892

[29] Benítez MA , Velasco C, Sequeira AR, Henríquez J, Menezes FM, Paolucci F. Responses to COVID-19 in five Latin American countries. Health Policy Technol 2020;9:525–59. 32874863 10.1016/j.hlpt.2020.08.014 PMC7451099

[30] R Core Team. *R: A Language and Environment for Statistical Computing.* R Foundation for Statistical Computing. 2020. http://www.R-project.org (28 January 2021, date last accessed).

[31] Cohen J , Kupferschmidt K. Countries test tactics in ‘war’ against COVID-19. Science 2020;367:1287–88. 32193299 10.1126/science.367.6484.1287

[32] Bi Q , Wu Y, Mei S et al Epidemiology and transmission of COVID-19 in 391 cases and 1286 of their close contacts in Shenzhen, China: a retrospective cohort study. Lancet Infect Dis 2020;20:911–19. 32353347 10.1016/S1473-3099(20)30287-5 PMC7185944

[33] Verity R , Okell LC, Dorigatti I et al Estimates of the severity of coronavirus disease 2019: a model-based analysis. Lancet Infect Dis 2020;20:669–77. 32240634 10.1016/S1473-3099(20)30243-7 PMC7158570

[34] Tang Y-W , Schmitz JE, Persing DH, Stratton CW. Laboratory diagnosis of COVID-19: current issues and challenges. J Clin Microbiol 2020;58:e00512–20. 32245835 10.1128/JCM.00512-20 PMC7269383

[35] Backer JA , Klinkenberg D, Wallinga J. Incubation period of 2019 novel coronavirus (2019-nCoV) infections among travellers from Wuhan, China. Euro Surveill 2020;25:2000062. 10.2807/1560-7917.ES.2020.25.5.2000062 PMC7014672 32046819

[36] Leon DA , Shkolnikov VM, Smeeth L, Magnus P, Pechholdová M, Jarvis CI. COVID-19: a need for real-time monitoring of weekly excess deaths. Lancet 2020;395:e81. 32333839 10.1016/S0140-6736(20)30933-8 PMC7176374

[37] Wu J , McCann A, Katz J, Peltier E. New York Times, 21 April 2020. *The Pandemic’s Hidden Toll: Half a Million Deaths*. https://www.nytimes.com/interactive/2020/04/21/world/coronavirus-missing-deaths.html (28 January 2021, date last accessed).

[38] The Economist, 15 July 2020. *Tracking COVID-19 Excess Deaths Across Countries*. https://www.economist.com/graphic-detail/2020/07/15/tracking-covid-19-excess-deaths-across-countries (28 January 2021, date last accessed).

[39] Mølbak K , Mazick A. European monitoring of excess mortality for public health action (EuroMOMO)Kåre Mølbak. Eur J Public Health 2013. doi: 10.1093/eurpub/ckt126.1133.

[40] The Human Mortality Database, University of California, Berkeley, Max Planck Institute for Demographic Research. *Short-term Mortality Fluctuations (STMF) Data Series*. 2020. http://www.mortality.org/ (28 January 2021, date last accessed).

[41] Institut national d’études démographiques. Demographics of COVID-19 Deaths. 2020. https://dc-covid.site.ined.fr/en/ (17 August 2020, date last accessed).

[42] Meslé F , Pison G; The Conversation. *Comment la France compte-t-elle ses morts? [How does France count its dead?]*. 2020. http://theconversation.com/comment-la-france-compte-t-elle-ses-morts-135586 (17 August 2020, date last accessed).

[43] Dong E , Du H, Gardner L. An interactive web-based dashboard to track COVID-19 in real time. Lancet Infect Dis 2020;20:533–34. 32087114 10.1016/S1473-3099(20)30120-1 PMC7159018

[44] Population Division, 2019, United Nations. World Population Prospects. https://population.un.org/wpp/ (17 August 2020, date last accessed).

